# A curious case of DBS radiofrequency programmer interference

**DOI:** 10.1038/s41531-019-0075-7

**Published:** 2019-02-01

**Authors:** Sanjeet S. Grewal, Karim ReFaey, Amy L. Grassle, Ryan J. Uitti, Robert E. Wharen

**Affiliations:** 1 Department of Neurological Surgery, Mayo Clinic, Jacksonville, FL USA; 2 Department of Neurology, Mayo Clinic, Jacksonville, FL USA

## Abstract

Deep brain stimulation (DBS) systems frequently rely on radiofrequency (RF) transmission for patient programming. The potential exists for other devices to interfere with communication between the internal pulse generator (IPG) and the programming device. In this paper, we are reporting a case of programming interference between the IPG and the WaveID device.

## Introduction

Deep brain stimulation (DBS) is an effective and safe treatment for movement disorders, such as Parkinson’s disease (PD), dystonia, and essential tremor.^1^ It functions by delivering an electrical stimulus to the targeted neuronal structures, such as basal ganglia and thalamic nuclei; the electrical stimulus is delivered through multi-contact intracranial electrodes. These electrodes are anchored to the skull and connected a subcutaneous pulse generator in the chest or the abdomen by extension. Disruption of the circuit can lead to hardware failure; one of the most common locations for circuit disruption is the extracranial portion of the DBS electrode and the extension wires.^2,3^ Although radiofrequency interference with DBS devices while approaching security, anti-theft, or radiofrequency identification (RFID) devices at the airport was described briefly,^4^ it was never reported in the literature prior. In this paper, we are reporting a case of programming interference between the internal pulse generator (IPG) and the WaveID device.

## Case presentation

**Fig. 1:**
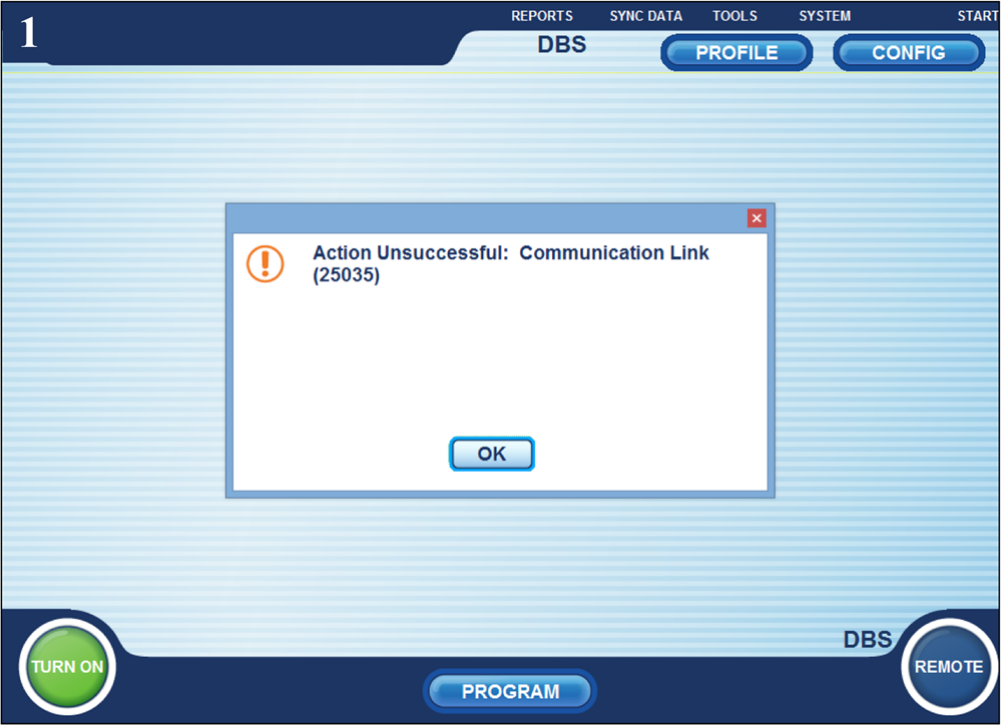
The error message: “action unsuccessful: communication link (25035)”

**Fig. 2:**
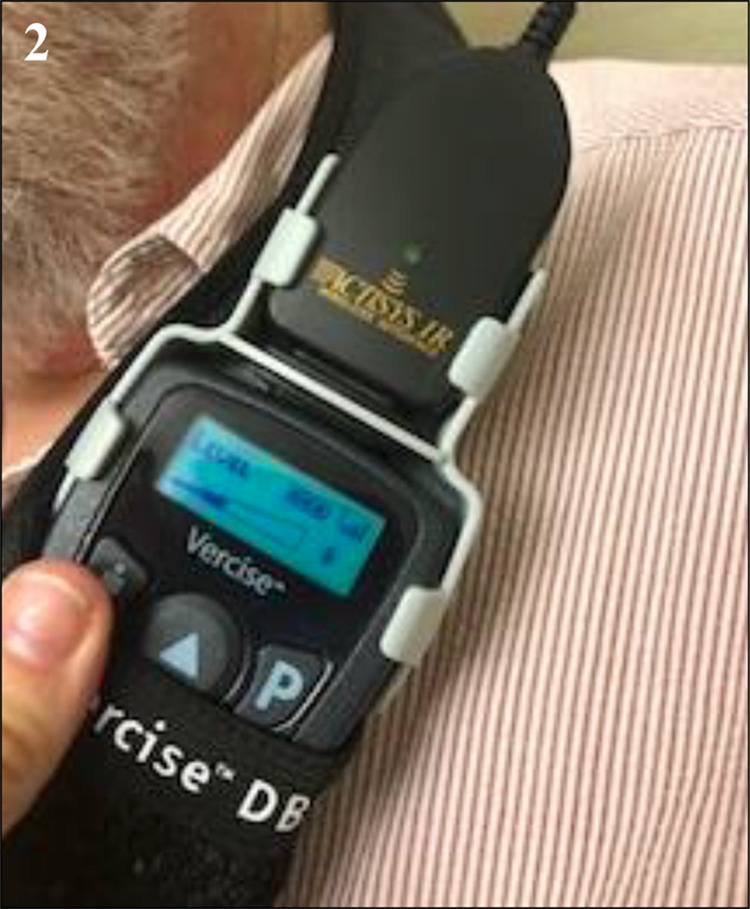
IPG showed a fully charged battery. Official permission was obtained to use Vercise brand name

**Fig. 3:**
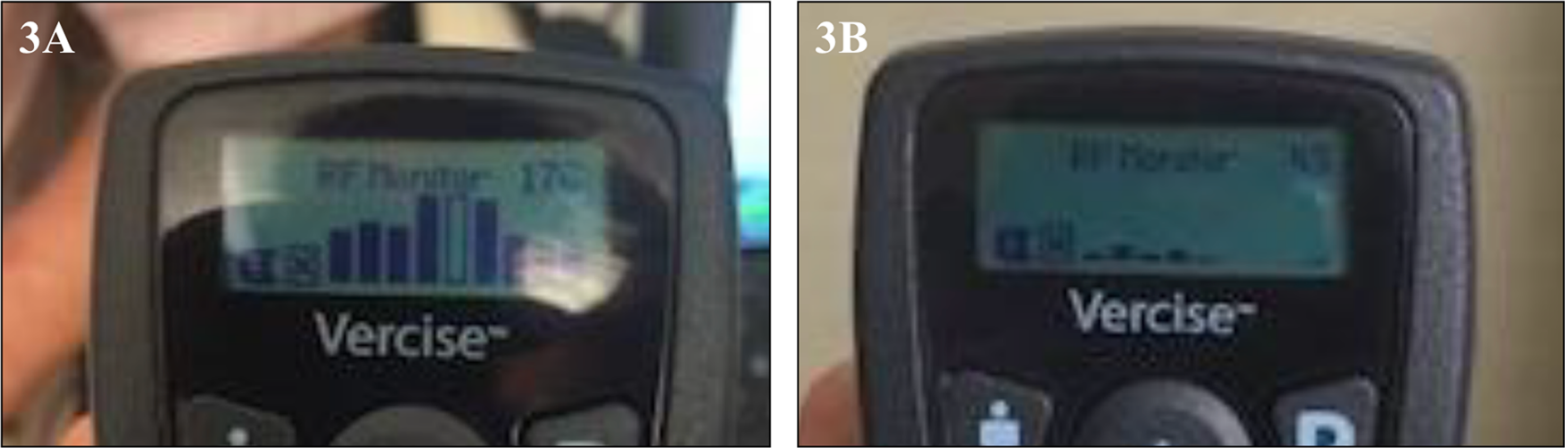
**a** RF reading at each corner in the room as well as where the patient was sitting (RF = 176). **b** The patient was moved to another spot in the room where the RF reading was lower (RF = 45). Official permission was obtained to use Vercise brand name

A 67-year-old gentleman with essential tremor underwent implantation of a Boston Scientific DBS system into the left VIM nucleus of the thalamus. At 3 months postoperatively he returned for modification of his programming due to increased tremor in his right hand. At this visit, there was an error in establishing communication between his IPG and the programming device. This patient had already been programmed at a different location and previously there had been no difficulties in connecting the programmer with his IPG. Troubleshooting of this issue began by replacing each component including the programming device, the connectors, and the computer one at a time, however, the patient’s device still did not connect. The computer would initiate data download from the device but an error message would appear at varying points during this process. The error message: “action unsuccessful: communication link (25035)” would be prompted **(**Fig. 1). This error message was noted to be a sign of radiofrequency interference. The following steps were then performed in an attempt to eliminate any interference: (1) ensure the IPG is fully charged **(**Fig. 2), (2) remove any power sources near the patient. RF readings were taken next to the patient and at every corner on the room. The patient location had an RF reading on 176 **(**Fig. 3a) compared to 87, 48, 67, and 78 for each of the corners. Within the patient remote control (RC) there is an option that measures RF. The RF meter is a standard “RSSI” (Received Signal Strength Indicator) indicator. Also, there is an ADC (analog-to-digital converter) that samples the signal as received by the receiver. The value of the ADC reading is then presented on the RC. The RC is held in the spot of interest for the measurement to be taken. The patient was then moved to a new location (RF reading at this new location was 45 (Fig. 3b), subsequently, the computer connected to the IPG and programming proceed normally. After completion, the patient was moved back to the original location and error message 25035 reappeared. The patient provided written informed consent.

## Discussion

**Fig. 4:**
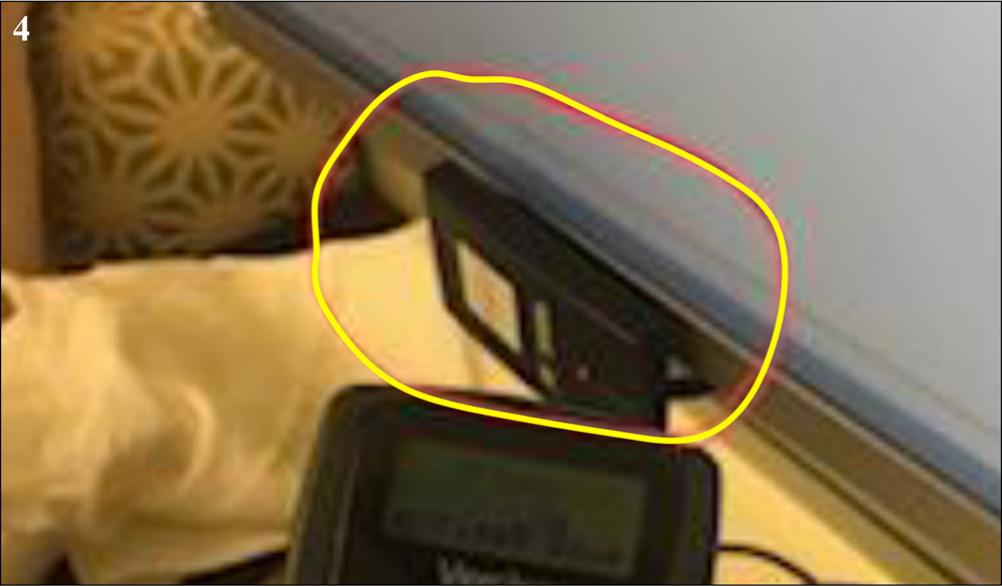
Showing the RF card (WaveID) card reader in the patient room that transmits at 125 kHz. Official permission was obtained to use Vercise brand name

Radiofrequency interference was initially described as electromagnetic interference (EMI), an electrical or magnetic field that prevents the neurostimulator from operating correctly.^5^ Currently, three companies are manufacturing DBS systems (Medtronic, St. Jude, and Boston Scientific). Each of the DBS systems transmits on a unique radiofrequency. Medtronic’s Activa DBS system transmits at a frequency of 175 kHz.^6^ St. Jude’s Infinity DBS systems transmits between 2.402 and 2.48 GHz.^7^ FCC search on the Boston Scientific Vercise remote indicates that it transmits at a frequency of 125 kHz.^8^ In this light, Boston Scientific has not altered their current generation product in any way. The best solution is to relocate the patient and card reader 4 feet apart. If this is not feasible, one might consider shielding the card reader. The FCC website was cross-referenced for devices that operate on a similar frequency range. It was discovered that the WaveID devices (manufactured by RF IDeas, Inc.) transmit at this frequency, and several RF card readers manufactured by RF Ideas were found to transmit at exactly 125 kHz (Fig. 4).^9^ WaveID is a badge-based authentication and identification solutions powered by RF IDeas readers, that enable employees to wave their badge for identification, Single-Sign-On in the medical and manufacturing industries, computer login, execute print jobs, meetings register at, track time and attendance, and pay for food.^10^ Additional research into the RF readers found that these systems are considered passive and constantly and continuously transmit at 125 kHz. This case highlights the possible effects of radiofrequency interference on DBS systems. Electronic devices, such as cell phones, WaveID readers that are present in the clinic rooms and hospitals, might cause additional interference; clinicians should be mindful of potential sources of interferences.

## Conclusion

The WaveID reader was a source of RF interference and prevented a connection between the IPG and programmer. RF interference is a potential source of difficulty in patient programming, as more and more devices are introduced to the clinic site, physicians must remain cognizant of these as potential sources of interference.

## Data Availability

Data are available on request from the authors.
